# Integrated analyses of lncRNAs microarray profiles and mRNA–lncRNA coexpression in smooth muscle cells under hypoxic and normoxic conditions

**DOI:** 10.1042/BSR20181783

**Published:** 2019-04-02

**Authors:** Qinshuo Zhao, Dating Sun, Yuanyuan Li, Jin Qin, JiangTao Yan

**Affiliations:** 1 Division of Cardiology, Departments of Internal Medicine, Tongji Hospital, Tongji Medical College, Huazhong University of Science and Technology, Wuhan 430030, People’s Republic of China; 2 Genetic Diagnosis Center, Tongji Hospital, Tongji Medical College, Huazhong University of Science and Technology, Wuhan 430030, People’s Republic of China

**Keywords:** atherosclerosis, bioinformatics analyses, hypoxia, LncRNA, mRNA, Smooth muscle cell

## Abstract

Hypoxia may cause abnormal proliferation and migration of the vascular smooth muscle cells (VSMCs) from the media to the intima. This contributes to vessel narrowing and accelerates the process of atherosclerosis. The association of the aberrant expression of long noncoding RNAs (lncRNAs) with the development and progression of atherosclerosis is well known; however, it is not well investigated in hypoxic VSMCs. Using a microarray approach, we identified 1056 and 2804 differentially expressed lncRNAs and mRNAs, respectively, in hypoxic and normoxic mouse aorta smooth muscle (MOVAS) cells. Of them, we randomly chose several lncRNAs and validated the microarray data using the quantitative PCR (qPCR) assay. Advanced bioinformatics analyses indicated that the up-regulated mRNAs were mainly involved in inflammatory responses, lipid metabolism, clearance of amyloid-β peptide, citrate cycle (TCA cycle), TGF-β signaling, and chemokine signaling. The down-regulated mRNAs were mainly involved in the apoptosis pathway, glycerolipid metabolism, Wnt signaling pathway, and MAPK signaling pathway. The constructed coexpression network indicated interactions between 87 lncRNAs and ten mRNAs. In addition, we demonstrated that the silence of lncRNA NONMMUT002434 expression could abrogate the migration and proliferation of smooth muscle cells dramatically. Our data provide comprehensive evidence on the differential expression of lncRNAs and mRNAs in hypoxic MOVAS cells, which may be valuable biomarkers for atherosclerotic diseases, and thereby facilitating diagnosis of atherosclerosis.

## Introduction

Atherosclerosis, a chronic progressive inflammatory disease of the arterial wall, is the primary etiology of various cardiovascular dysfunctions, including unstable angina, myocardial infarction (MI), heart failure, and aneurysm [1]. Atherosclerosis is increasingly becoming a significant cause of morbidity and mortality worldwide [2]. VSMCs are the prime constituents of the foam cell population in atherosclerotic plaque and play a major role in the pathogenesis of atherosclerosis [3]. Moreover, the inflammation and apoptosis of VSMCs are responsible for the rupture and instability of plaque. In addition, the uncontrolled proliferation of VSMCs promotes plaque formation and atherogenesis which aggravate the restenosis of the vascular lumen [4]. Upon vascular injury, VSMCs migrate into the intima of vessels, acquire an inflammatory phenotype, and produce extracellular matrix in the plaque area. This is the central axiom of most models of atherosclerosis [5, 6]. Previous studies had indicated that hypoxia was a vital factor in atherosclerotic cardiovascular disease, which increased the uptake and infiltration of labeled low-density lipoprotein in VSMCs [7]. Hypoxia has been suggested to accelerate atherosclerosis by worsening lipid metabolism and increasing inflammatory responses [8–10]. In addition, HIF-1α promotes cellular lipid aggregation [11] and hypoxia-inducible protein 2 participation in lipid droplet formation [12]. In atherosclerosis, VSMCs are activated to undergo proliferation and migration that contribute to vascular lumen narrowing and local hypoxia [13]. In turn, hypoxia may lead to the dysfunction of various components of atherosclerotic plaque by promoting lipid accumulation and foam cells formation [14, 15]. Interestingly, recent studies have indicated that hypoxia-associated diseases including obstructive sleep apnea, which is characterized by hypoxia in sleep, are independently linked with atherosclerosis [16, 17]. Therefore, extensive systematic laboratory and clinical studies are needed to explore the pathogenesis of hypoxia in atherosclerosis in detail.

Long noncoding RNAs (lncRNAs), transcripts that are longer than 200 nts with little or no protein-coding capacity, constitute a large part of the human genome [18, 19]. The major role of lncRNAs is to serve as a mediator in various signaling pathways involved in cellular proliferation, differentiation, apoptosis, and organ development [20–22]. They also act as valuable biomarkers in cardiovascular diseases. For example, lncRNA *Chrf* has been shown to be significantly up-regulated in a transaortic constriction mouse model, suggesting that it might be a promising biomarker of cardiac hypertrophy [23]. Additionally, lncRNA *MIAT* was found to be highly expressed in injured heart tissues and linked to high risk of MI, based on a large-scale case-control association study [24]. Interestingly, recent studies have revealed that lncRNAs could affect the process in atherosclerosis, including regulation of the proliferation and migration of VSMCs [25–27], lipid metabolism [28], and the associated vascular immune and inflammatory responses [29–31]. However, the underlying molecular mechanisms as well as the lncRNA biomarkers in the VSMCs exposed to hypoxia are yet to be identified. Therefore, building a comprehensive understanding of the dysregulation of lncRNAs in the VSMCs exposed to hypoxia is essential for patients diagnosed with atherosclerotic diseases.

In the current study, we investigated the expression profiles of lncRNA and mRNA in MOVAS cells under hypoxic and normoxic conditions to understand the pathogenesis of atherosclerosis.

## Methods and materials

### Cell culture

The mouse aorta smooth muscle (MOVAS) cell line was purchased from iCell Bioscience lnc (Shanghai, China). The cells were cultured on uncoated polystyrene dishes in Dulbecco’s modified Eagle’s medium (Gibco) supplemented with 10% FBS (Gibco, Invitrogen, Grand Island, NY, U.S.A.) and maintained in a humidified atmosphere of 95% air and 5% CO_2_ at 37°C. For subculturing, the viable adherent cells were detached using 0.25% trypsin containing 1 mM EDTA at 37°C for approximately 1 min. The dispersed cells were then reseeded in six-well plates at a density of approximately 3 × 10^5^ cells/well. In order to create hypoxic condition, the cultured cells were administrated cobalt dichloride (CoCl_2_, 200 µM, Sigma, U.S.A., dissolved in water) exogenously and cultivated for 24 h.

### Western blot analysis

Proteins were extracted from the whole cultured cells. The protein concentrations were measured using a BCA Protein Assay Kit (Boster, Wuhan). Equal quantities of protein samples were subjected to 10% SDS/PAGE and then transferred to PVDF membranes; the housekeeping protein, antiglyceraldehyde-3-phosphate dehydrogenase (GAPDH) was used as the control. The antibodies, rabbit polyclonal anti-HIF1-α antibody (1:1000 dilution, Abcam, U.S.A.) and mouse monoclonal GAPDH antibody (1:1000 dilution, CST, U.S.A.) were used in this experiment.

### Quantitative real-time PCR

Total RNA was extracted using TRIzol regent (Invitrogen) from the cultured MOVAS cells. The concentration and quality of total RNA were measured using Nanodrop spectrophotometer (ND-1000, Nanodrop Technologies). The cDNA first strands were synthesized using the SuperScript^®^III First Strand Synthesis Kit (Life Technologies, Carlsbad, CA, U.S.A.) according to the manufacturer’s protocol. The selected lncRNAs of the primers used for real-time PCR (RT-PCR) were synthesized by Tianyibiotech (Wuhan, China), and the information of which were showed in Supplementary Table 3. RT-PCR assays were performed using the SYBR® Select Master Mix (Thermo scientific, U.S.A.) on a 7900HT FAST Real-Time PCR System (Life Technologies, Carlsbad, CA, U.S.A.). All data were subsequently normalized to the GAPDH mRNA level, and the fold change of lncRNA was calculated using the 2^−ΔΔ*C*^_T_ quantitation, each qPCR reaction was run three separate times.

### Microarray data analysis

Four pairs of samples were prepared for the lncRNA and mRNA microarray data analyses using Agilent Feature Extraction software (version 11.0.1.1, Agilent Technology). The slides were washed in staining dishes with a Gene Expression Wash Buffer Kit (Agilent, Santa Clara, CA, U.S.A.) and scanned using an Agilent Microarray Scanner (Agilent, Santa Clara, CA, U.S.A.) with default settings according to the manufacturer’s instructions. Following quantile normalization and subsequent raw data, the processing of lncRNA and mRNA data was performed using the R software package, limma (Agilent Technologies). The differentially expressed lncRNAs and mRNAs were identified using R software (version 3.2.3) [32]. The lncRNAs and mRNAs were carefully collected from reliable data sources, including GENCODE, Ensembl, LNCipedia, Lncrnadb, Noncode, and UCSC. The differentially expressed lncRNAs and mRNAs with a statistically significant difference in expression between the two groups were confirmed through the *P*-value, false discovery rate (FDR), and fold change filtering (2-fold changes in expression level and differences with FDR < 0.05 were considered significant). The *P*-value was adjusted by the Benjamini-Hochberg method. In order to determine the up-regulated and down-regulated lncRNAs and mRNAs, significant threshold values were considered to be a fold change (linear) ≥ 2.0 or ≤ 0.5 along with *P*≤0.05.

### Bioinformatic analysis

Gene ontology (GO) analyses (http://www.geneontology.org) provide three structured networks that describe the genes and gene product properties in terms of cellular components, molecular functions, and biological processes [33, 34]. The *P*-value denotes the significance of the GO-term enrichment in the list of differentially expressed mRNAs, and its cutoff was set at 0.05. Pathway analyses and functional analyses that analyze the potential functions of the differentially expressed genes in the pathways were carried out using the most recent Kyoto encyclopedia of genes and genomes (KEGG) database. The *P*-value denotes the significance of the pathway correlated to the conditions, and its cutoff was also set at 0.05.

### LncRNA/mRNA coexpression network

To investigate the relationship between lncRNAs and mRNAs, we constructed the lncRNA–mRNA correlation network. The data were first filtered using the median gene expression value of all lncRNAs. Then, we screened the data for differentially expressed lncRNAs and retained qualified data based on the reliability of the sample signal and lncRNA target prediction dataset. Finally, we screened for meaningful data based on the Pearson correlation coefficient (which is equivalent to or larger than 0.9) and constructed the coexpression network using Cytoscape software (Version3.5.1).

### siRNA transfection and transwell migration assay

The siRNA of the lncRNA, NONMMUT002434 and the corresponding scramble negative control siRNA were designed by Ribobio (Guangzhou, China). We used three specific siRNAs for silencing the corresponding target of interest and mixed them for transfection. Information on the siRNAs used is provided in Supplementary Table 4. The negative control siRNA was also transfected under identical conditions for comparison. The siRNAs were transfected into MOVAS cells at a final concentration of 50 nM using lipofectamine^®^2000 Reagent (Invitrogen) according to the manufacturer’s instruction. After transfection for 48 h, the cells were harvested for further assays. The inhibition efficiency of siRNA was confirmed by q-PCR analyses.

For the cell migration assay, approximately 1 × 10^5^ cells were seeded into the upper chamber of a transwell insert (8.0 μm; Millipore, Billerica, MA, U.S.A.) containing 100 μl serum-free culture medium in each well. Concurrently, the lower chambers were filled with 500 μl culture medium containing 10% serum as a chemoattractant. The seeded cells were incubated for 12 h to facilitate migration. Subsequently, the cells in the upper part of the filter were discarded using small cotton swabs, and the cells on the lower surface of the insert were washed thrice with 1 × PBS, fixed with 4% formaldehyde (Sigma), and stained with 0.1% crystal violet for 20 min. The transmigrated cells were counted manually under an inverted microscope and their images were captured.

### CCK8 and EdU assay

Cell proliferation was assessed using the cell counting kit-8 and the EdU incorporation assay. For CCK-8 evaluation, the transfected cells were seeded in 96-well plates at a density of approximately 4 × 10^3^ cells/well. The assays were performed 1 to 3 days post-plating following the addition of 100 μl fresh medium containing 10 μl of CCK-8 solution and incubation for another 4 h at 37°C. The absorbance of the solution in each well was detected at 450 nm using a Synergy2 spectrometer. The assay was repeated in triplicate.

For the EdU assay, transfected MOVAS cells were plated in 24-well plates at about 4 × 10^4^ cells/well. The cells were incubated with 100 μM EdU for 2 h before fixation with 4% paraformaldehyde, permeabilization with 0.5% Triton X-100 and were finally subjected to EdU staining. Consequently, the cells were washed thrice with PBS, stained with 5 μg/ml DAPI (4′,6-diamidino-2-phenylindole) for 5 min and immediately viewed under a fluorescence microscope. The number of EdU-positive cells at three random fields was counted.

### Statistical analysis

All data are presented as mean ± SEM. Statistical analyses were performed using GraphPad Prism 5 (GraphPad Software, San Diego, CA, U.S.A.). The statistical significance was assessed by two-tailed unpaired *t*-test for two groups or by multiple comparison ANOVA for more than two groups (Bonferroni’s multiple comparisons test, *P*<0.05). Genes with a two-sided *P*-value < 0.05 and fold change > 2.0 or < 0.5 were defined as statistically significant differentially expressed genes.

## Results

### Identification of differentially expressed lncRNAs and mRNAs

**Figure 1:**
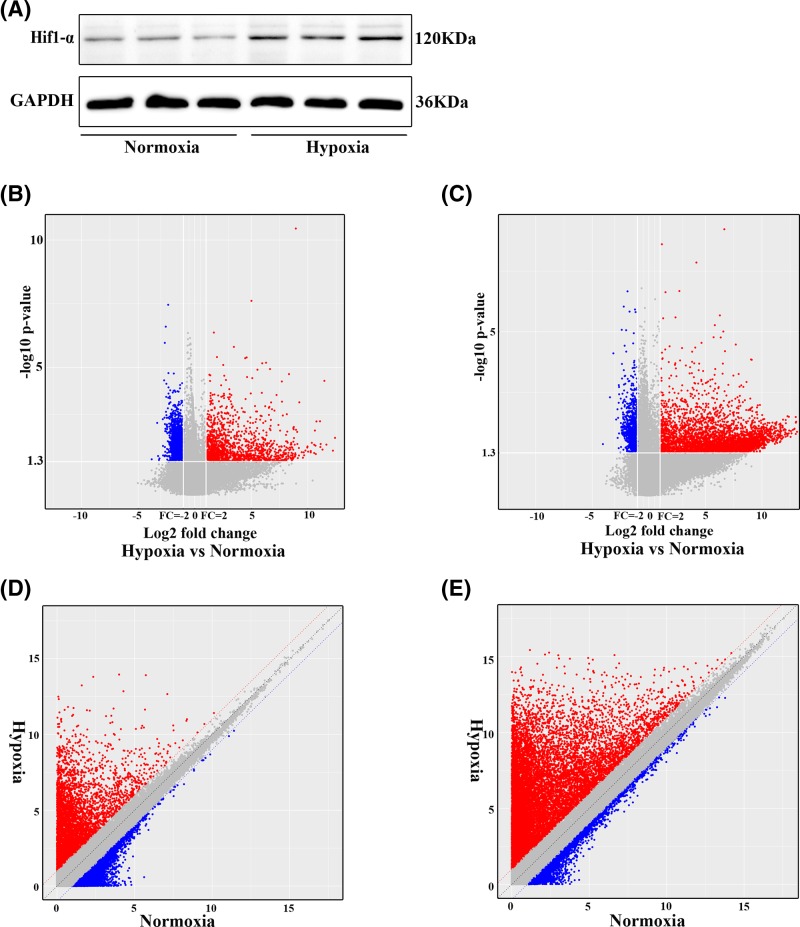
LncRNA and mRNA expression profiles in hypoxic and normoxic MOVAS cells (**A**). Detection of HIF1-α expression using Western blotting assay. Scatter plots were used to distinguish the differentially expressed (**B**) lncRNA and (**C**) mRNA between hypoxic and normoxic groups, respectively. (**D**) and (**E**) Volcano plots of lncRNAs and mRNA expression levels between the hypoxic and normoxic groups, respectively. The dendrogram demonstrates the relationships of the lncRNA and mRNA expression profiles between the hypoxia and normoxia MOVAS cell samples, The red dots represent up-regulated lncRNAs and mRNAs, and the green dots represent the down-regulated lncRNAs and mRNAs, respectively (*P*<0.05; fold change > 2.0).

**Figure 2:**
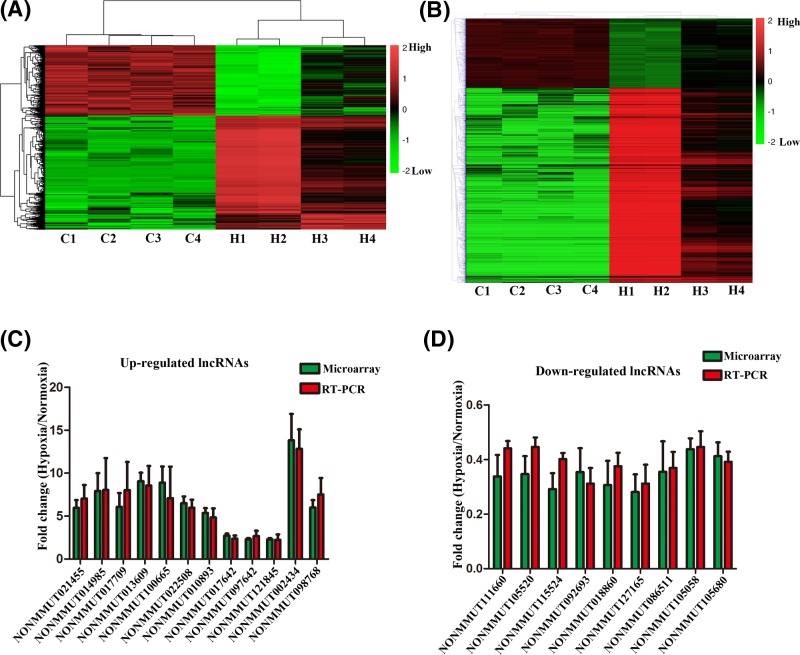
Heat map of differentially expressed lncRNAs and mRNAs of MOVAS cells and validation of the microarray results by RT-qPCR Hierarchical clustering analyses of (**A**) lncRNAs and (**B**) mRNAs. C1–C4 represent normoxic samples; H1–H4 represent hypoxic samples. The color scale on the right illustrates the relative expression level of lncRNAs and mRNAs across all samples. The red shades indicate high relative expression, and the green shades indicate low relative expression. (**C**) and (**D**) The levels of up-regulated lncRNAs and down-regulated lncRNAs were detected using RT-qPCR. All data are representative of three independent experiments.

First, we confirmed the hypoxic condition in the MOVAS cells treated with exogenous cobalt dichloride by Western blotting assay. The results showed that the expression of HIF1-α increased considerably in the hypoxic group compared with that of the control group (Figure 1A). Subsequently, to study the potential role of lncRNAs in atherosclerosis, we performed microarray analyses and determined the lncRNA and mRNA expression profiles of MOVAS cells under hypoxic and normoxic conditions. We hence identified 1056 lncRNAs and 2804 mRNAs to be differentially expressed. Of those, we found 644 up-regulated and 412 down-regulated lncRNAs, and 2034 up-regulated and 770 down-regulated mRNAs (fold change > 2.0 and < 0.5 for up-regulation and down-regulation, respectively, *P*<0.05). The volcano plot and scatter analyses were then employed for the direct identification of differences in lncRNAs (Figure 1B,C) and mRNAs (Figure 1D,E). Based on the expression levels of the lncRNAs and mRNAs from the microarray analyses, we created a heat map of differentially expressed lncRNAs (Figure 2A) and mRNAs (Figure 2B) in the hypoxic and normoxic MOVAS cells. The top 30 dysregulated lncRNAs and mRNAs are listed in Supplementary Table 1 and 2, respectively.

### Validation of lncRNAs by RT-qPCR

In order to validate our results, we randomly selected 14 up-regulated and 12 down-regulated differentially expressed lncRNAs to perform q-PCR. The PCR results confirmed the dysregulated expression of 12 up-regulated (Figure 2C) and nine down-regulated lncRNAs (Figure 2D) that were identified. The lncRNA, NONMMUT002434 showed the most elevated expression (18.1-fold higher expression), and NONMMUT115524 showed the lowest expression (0.39-fold lower expression). These results were analogous to those obtained from the microarray chip data.

### Examining the functions of the differentially expressed genes

**Figure 3:**
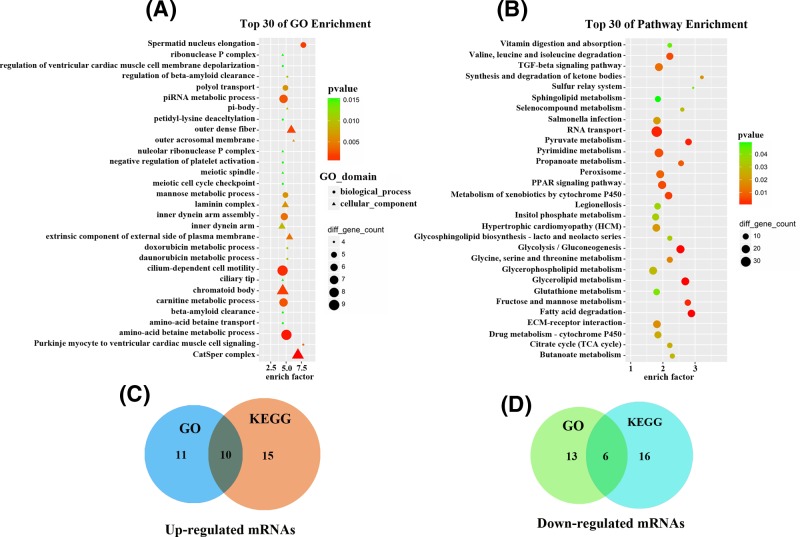
Gene ontology and Kyoto Encyclopedia of Genes and Genomes pathways analyses of differentially expressed mRNAs (**A**) The top 30 dysregulated genes from GO enrichment analyses that are related to biological processes (BP), cellular components, (CC) and molecular functions (MF). (**B**) The top 30 dysregulated genes from the KEGG pathway enrichment analyses correlated with cellular processes, metabolism, and environmental information processes, (**C**) and (**D**) Venn diagram of the common differentially expressed genes which are correlated with atherosclerosis according to GO and KEGG pathway enrichment analyses with the overlap of 16 genes (ten up-regulated genes; six down-regulated genes).

To evaluate the biological roles of the mRNAs expressed in the VSMCs exposed to hypoxia, GO enrichment and KEGG pathway analyses were performed. The GO analyses showed that the up-regulated mRNAs were mainly associated with the response to laminin complex (GO: 0043256), glycerol transmembrane transporter activity (GO: 0015168), positive regulation of cholesterol esterification (GO: 0010873), clearance of amyloid-β peptide (GO: 0097242) and out dense fiber (GO: 0001520), while the down-regulated mRNAs were involved in angiogenesis (GO: 0001525), electron transport chain (GO: 0022900), membrane lipid metabolic process (GO: 0006643) and cell–cell adhesion (GO: 0098609) (Figure 3A). Subsequent pathway analyses indicated that up-regulated mRNAs were involved in inflammatory responses, lipid metabolism, clearance of amyloid-β peptide, citrate cycle (TCA cycle), TGF-β signaling, and chemokine signaling pathway, while the down-regulated mRNAs were involved in adherens junction, apoptosis pathway, glycerolipid metabolism, Wnt signaling pathway, and MAPK signaling pathway (Figure 3B). These data suggest that these GO term-associated functions and KEGG pathways may contribute to the pathogenesis of atherosclerosis when the VSMCs are in hypoxic condition.

With the GO terms enrichment, 21 up-regulated and 19 down-regulated mRNAs were predominant, whereas 25 up-regulated and 22 down-regulated mRNAs were predominant with the KEGG pathway enrichment owing to their association with atherosclerosis. Finally, 16 mRNAs (ten up-regulated and six down-regulated) overlapped in both the GO enrichment and KEGG pathway analyses results (Figure 3C,D). Of those, ten mRNAs were associated with 124 differentially expressed lncRNAs.

### LncRNA–mRNA coexpression network

**Figure 4:**
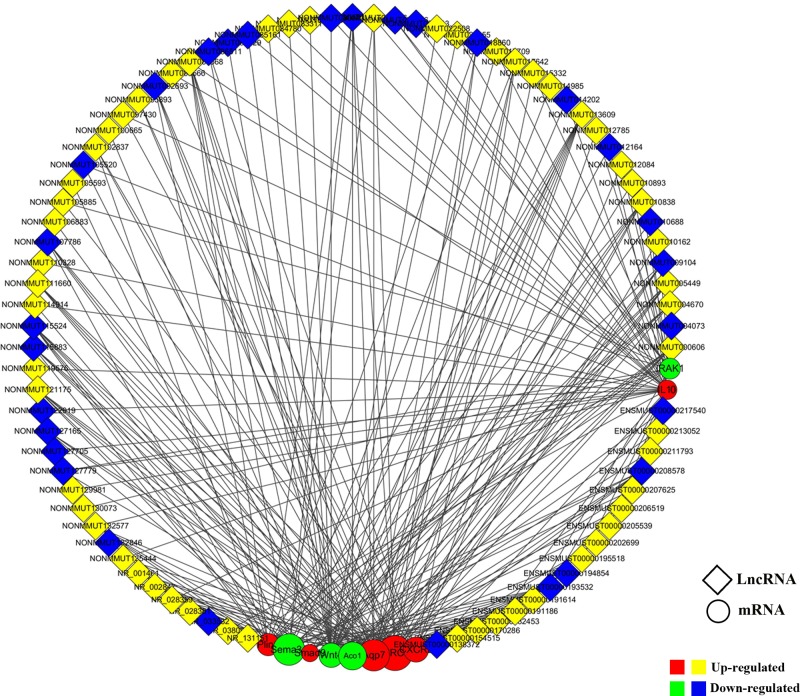
Construction of the lncRNA–mRNA coexpression network Coexpression network analyses were performed to characterize the 87 differentially expressed lncRNAs and ten differentially expressed mRNAs (six up-regulated and four down-regulated) based on the microarray results using Cytoscape 3.5.1 software. Red circular nodes indicate up-regulated mRNAs, and green circular nodes indicated down-regualted mRNAs. Yellow rhombus nodes denote up-regulated lncRNAs, whereas blue rhombus nodes designate down-regulated lncRNAs.

A coexpression network analysis was performed to characterize the 124 differentially expressed lncRNAs and the associating ten overlapping mRNAs (six up-regulated and four down-regulated). They were selected to construct the lncRNA–mRNA coexpression network using Cytoscape software. Our data showed that a total of 87 lncRNAs (58 up-regulated and 29 down-regulated) and ten mRNAs were connected with 226 coexpression edges. Of those, 14 lncRNAs interacted with one mRNA (*Smad9*) in the TGF-β signaling pathway, 92 lncRNAs interacted with four mRNAs (*CXCR2, IL10, Wnt4*, and *IRAK1*) that are associated with inflammatory response, 93 lncRNAs interacted with four mRNAs (*Sema3e, Apq7, Aco1*, and *Plin3*) that are related to lipid metabolism and 27 lncRNAs interacted with one mRNA (*Birc5*) that is related to antiapoptosis (Figure 4).

### Effect of NONMMUT002434 on migration and proliferation *in vitro*

**Figure 5:**
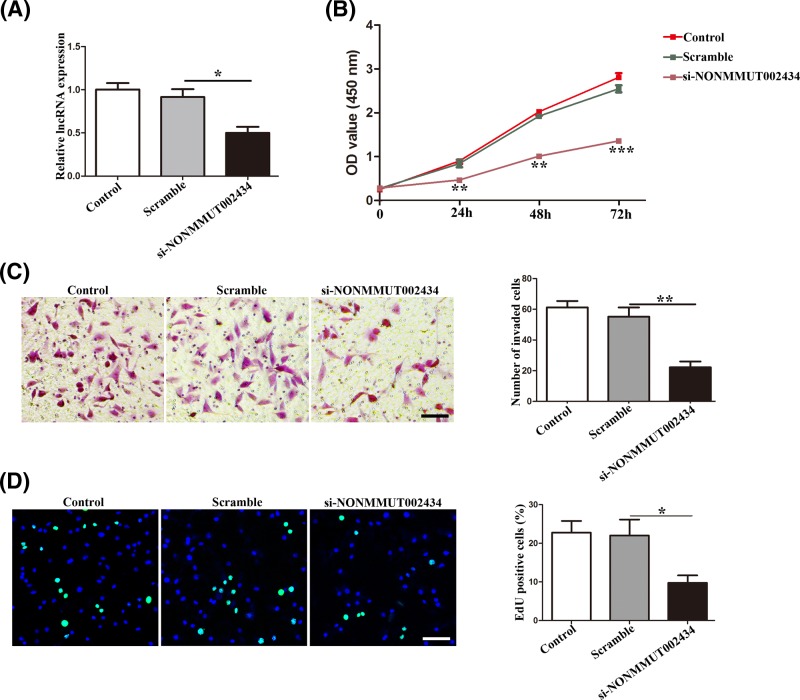
Effect of NONMMUT002434 knockdown on MOVAS cells invasion, proliferation (**A**) The expression of NONMMUT002434 after siRNA transfection. (**B**) CCK-8 proliferation, n = 5 per group, scramble group versus si-NONMMUT002434 group. (**C**) The migratory and invasive ability of MOVAS cells after knockdown of NONMMUT002434, assessing by transwell assay. Scale bar, 100 μm in each graph. The right graph represents quantitation of invaded cells. (**D**). Representative images showing EdU fluorescence staining to detect the newly synthesized DNA. Blue fluorescence shows cell nuclei and green fluorescence represents cells with DNA synthesis. Scale bar, 100 μm in each graph. The right graph shows the percentage of EdU-positive cells, calculating using ImageJ software, scar bar = 100 um. Data are expressed as mean ± S.D.; * *P*<0.05; **P<0.01; ***P<0.001. Similar results were obtained from experiments with three replicates.

To elucidate the possible role of lncRNAs in atherosclerosis, we employed lncRNA-specific siRNA-targetted NONMMUT002434 silencing in MOVAS cells under the normoxic condition, based on above coexpression, RT-PCR, and lncRNA microarray results for the sample detection and determination. First, we confirmed that the expression of the lncRNA, NONMMUT002434 was suppressed considerably in the MOVAS cells when transfected with si-NONMMUT002434, compared with that of the scrambled siRNA (Figure 5A). The CCK8 assay revealed that knockdown of the lncRNA, NONMMUT002434 could inhibit the proliferation of MOVAS cells (Figure 5B). Consistent with these results, the transwell assay indicated that the silencing of the lncRNA, NONMMUT002434 inhibited the invasive ability of the MOVAS cells (Figure 5C). Similarly, the EdU incorporation assay indicated that knockdown of the lncRNA, NONMMUT002434 could abrogate the proliferation of MOVAS cells (Figure 5D). These results indicate that the lncRNA, NONMMUT002434 may be involved in the progression of atherosclerosis, and inhibiting its expression may protect VSMCs from atherosclerotic progression.

## Discussion

VSMCs are thought to be the key players in atherosclerotic lesion progression and restenosis. The balance of VSMCs proliferation and migration versus VSMCs necrosis and apoptosis determines the fate of the atherosclerotic plaque [3]. Increasing evidence shows that the state of hypoxia may promote the progression of atherosclerosis by aggravating extracellular lipid deposits and inflammatory responses, thus contributing to the plaque instability [35]. However, little is known about the relationship between the differential expression of lncRNAs in VSMCs and atherosclerosis under hypoxic condition. We speculated that lncRNAs are potential regulators of atherosclerosis progression when VSMCs were subjected to hypoxia. Therefore, we performed transcriptomic profiling of the lncRNAs and protein-coding genes in VSMCs using a microarray platform. We selected MOVAS cell lines as our experimental subject, in which host lncRNAs and mRNAs were extensively investigated.

The present study is intriguing from different perspectives. First, we built intact lncRNA and mRNA expression profiles of MOVAS cells under hypoxic and normoxic conditions. Our results revealed 1056 differentially expressed lncRNAs and 2804 differentially expressed mRNAs. Of those, 644 up-regulated and 412 down-regulated lncRNAs, 2034 up-regulated and 770 down-regulated mRNAs were identified. The results showed strong correlations between the expression of lncRNAs in RT-qPCR analyses and microarray data analyses, and this confirmed the validity of our observations. Following that, GO and KEGG pathway analyses were performed to identify the detailed biological functions and potential mechanisms of these lncRNAs and mRNAs. We found that the up-regulated mRNAs were mainly associated with the response to laminin complex, glycerol transmembrane transporter activity, regulation of cholesterol esterification, clearance of amyloid-β peptide and out dense fiber. Meanwhile, the down-regulated mRNAs were enriched in angiogenesis, electron transport chain, membrane lipid metabolic process, and cell-cell adhesion. Subsequent pathway analysis indicated that up-regulated mRNAs were involved in inflammatory responses, lipid metabolism, the clearance of amyloid-β peptide, citrate cycle (TCA cycle), TGF-β signaling, and chemokine signaling pathway. In contrast, the down-regulated mRNAs were involved in apoptosis pathway, glycerolipid metabolism, Wnt signaling pathway, and MAPK signaling pathway. The results indicate that the inter-regulation of lncRNAs and mRNAs may be involved in the progression of atherosclerosis. Further, to confirm their relationship and the underlying regulatory mechanism of these lncRNAs and mRNAs, we constructed an lncRNA–mRNA coexpression network. It indicated that 14 lncRNAs interacted with one mRNA (*Smad9*) in the TGF-β signaling pathway, 92 lncRNAs interacted with four mRNAs associated with inflammatory responses (*CXCR2, IL10, Wnt4*, and *IRAK1*), 93 lncRNAs interacted with four mRNAs (*Sema3e, Aqp7, Aco1*, and *Plin3*) related to lipid metabolism, and 27 lncRNAs interacted with one mRNA (*Birc5*) related to antiapoptosis.

There are several well-accepted molecular mechanisms that have been reported to be involved in the development and progression of atherosclerosis, including the activation of the TGF-β signaling pathway, inflammation response, lipid metabolism, and apoptosis. TGF-β modulates the fibrotic components of lesion, and disruption of TGF-β signaling in VSMCs accelerates lesion progression and stimulates VSMCs proliferation [36]. Moreover, inflammatory cytokines induce the apoptosis and death of SMCs, which triggers negative vascular remodeling, calcification and massive infiltration of the macrophages *in vivo* [37]. Using apolipoprotein E(-/-) mice as a model, *in vivo* evidence also showed that VSMC apoptosis accelerates plaque growth and increased plaque instability during atherogenesis and in established plaques [38]. Lipid metabolism is fairly associated with atherosclerosis, oxidized LDL (oxLDL) and redundant cholesterol accumulation critically contribute to lesion growth and accelerate the progression of atherosclerosis [39]. Besides, oxLDL promote inflammation and induce endothelial dysfunction by producing a group of bioactive molecules [40]. Therefore, interventions developed to target these key pathogenic molecules may result in effective treatments and prevention of atherosclerotic diseases.

From the coexpression networks analyses, we found that NONMMUT002434 showed a high correlation with *IL10, Wnt4, Sema3e*, and *BIRC5* mRNA. The product of *IL10* is an anti-inflammatory cytokine, while *BIRC5* encodes a protein, which negatively regulates apoptosis. Thus, we inferred that NONMMUT002434 promote atherosclerosis by inhibiting IL10 and BIRC5, thus promoting inflammation and cell apoptosis. Therefore, we surmised that the lncRNA, NONMMUT002434 is likely to play an important role in inflammatory responses, lipid metabolism, and apoptosis in the progression of atherosclerosis under hypoxic conditions. In the current study, we transfected the corresponding siRNA of the lncRNA, NONMMUT002434 into the cultured cells under normoxia, and observed that the knockdown of the lncRNA, NONMMUT002434 resulted in decreased MOVAS cell proliferation and migration.

Numerous studies have confirmed that lncRNAs can be up-regulated or down-regulated by hypoxia, and that hypoxia plays important roles in modulating the formation of atherosclerotic plaque. For instance, the lncRNA HOTAIR and *UCA1* were induced by hypoxia through HIF-1a and induced cell proliferation, and migration [41, 42]. For this reason, we believe that lncRNAs may contribute to the regulation of hypoxia-induced atherosclerotic pathogenesis. To the best of our knowledge, this is the first report where lncRNAs and mRNAs are completely profiled in the VSMCs exposed to hypoxia, and the lncRNA, NONMMUT002434 is a novel lncRNA which has not been reported formerly. Several studies have also investigated whether lncRNAs are capable of regulating proliferation, migration and matrix synthesis in VSMCs, thereby affecting the development of atherosclerosis [25, 43, 44]. However, these studies were imperfect as they mainly focussed on the regulation and alteration of lipid protein and cholesterol levels induced by lncRNAs and ignored the impact of hypoxia on VSMCs. Our study will help us profoundly to understand the pathogenic effect and progress of hypoxia in atherosclerosis.

Despite these meaningful results, there were also some limitations in our study. First, due to the small sample size, it was difficult for us to determine the exact role of hypoxia on the induced lncRNAs in VSMCs. Second, further *in vivo* studies are needed to figure out the concrete mechanism behind the phenomenon of hypoxia-induced lncRNAs influencing the progress of atherosclerosis. The present study reports only a preliminary work and more extensive research remains to be carried out.

In summary, we first established the expression profiles of the lncRNAs and mRNAs in VSMCs exposed to hypoxia using a microarray approach. In addition, by means of bioinformatics tools, we found an undiscovered lncRNA, NONMMUT002434, which may participate in the proliferation and migration of VSMCs, and thus may be involved in the progression of atherosclerosis. As the effects of lncRNAs in atherosclerosis have not yet been fully understood, the present study may serve as valuable resource for further research.

## Supporting information

**Supplementary Table 1:** Top 30 up-regulated lncRNAs

**Supplementary Table 2:** Top 30 down-regulated lncRNAs

**Supplementary Table 3:** Sequences of PCR primers used for amplification of lncRNAs.

**Supplementary Table 4:** Information of siRNAs sequences for transfectionof.

## Abbreviations

CHRF: cardiac hypertrophy related factor
CXCR2: CXC motif chemokine receptor 2
FDR: false discovery rate
GAPDH: antiglyceraldehyde-3-phosphate dehydrogenase
GO: gene ontology
IL10: interleukin-10
KEGG: kyoto encyclopedia of genes and genomes
LncRNA: long noncoding RNA
MI: myocardial infarction
MIAT: MI associated transcript
MOVAS: mouse aorta smooth muscle cell
OSA: obstructive sleep apnea
oxLDL: oxidized LDL
qPCR: quantitative polymerase chain assay
VSMC: vascular smooth muscle cell
