# Supplementary material for: Integrated analyses of lncRNAs microarray profiles and mRNA–lncRNA coexpression in smooth muscle cells under hypoxic and normoxic conditions
Source: Biosci Rep. 2019 Apr 2;39(4):BSR20181783. doi: 10.1042/BSR20181783 (PMC6443952; doi:10.1042/BSR20181783)
Supplement: Supplementary file 1 [file bsr20181783_Supp1.pdf]

Supplementary Table 1.Top 30 up-regulated lncRNAs

LncRNA_ID	Length (bp)	Normoxia group	Hypoxia group	Log2FC	Pvalue
NONMMUT018986.1	2054	3.42	7.89	25.8244	0.0013
NONMMUT002434.1	2866	4.22	7.87	18.07	0.0167
NONMMUG014815.1	1557	4.67	8.06	17.12	0.0310
NONMMUG041586.1	1326	4.37	7.57	16.10	0.0467
NONMMUT117869.1	850	3.75	6.79	13.04	0.0380
NONMMUT112794.1	3760	3.63	6.69	11.43	0.0218
NONMMUT002084.1	1847	2.37	5.64	11.39	0.0319
NR_015613.1	2941	3.61	6.41	10.78	0.0468
NONMMUT128850.1	681	2.70	5.16	9.01	0.0112
NONMMUT094293.1	950	3.64	6.51	8.17	0.0048

NONMMUT024146.1	826	3.38	5.84	6.97	0.0249
NONMMUT020687.1	1508	3.71	6.01	6.73	0.0437
NONMMUT121845.1	525	3.95	6.57	6.64	0.0028
NONMMUG076404.1	525	3.96	6.58	6.64	0.0028
ENSMUSG00000087495	1073	3.95	6.26	6.48	0.0370
NONMMUG077390.1	1601	4.33	6.77	6.43	0.0160
NONMMUG014406.2	1515	4.72	6.96	6.14	0.0390
NONMMUG061752.1	635	3.96	6.14	5.99	0.0001
ENSMUSG00000110670.1	8757	4.44	6.55	5.64	0.0001
ENSMUSG00000108387.1	235	4.83	6.87	5.36	0.0470
NONMMUG053745.1	4467	3.19	5.60	5.01	0.0046
ENSMUSG00000107964.1	928	3.96	5.84	4.64	0.0490
NONMMUG084694.1	1094	5.86	7.86	4.42	0.0120
ENSMUSG00000106122.1	650	7.18	9.04	4.39	0.0380
NONMMUG001028.2	2498	5.28	7.14	4.35	0.0370

NONMMUG083485.1	650	4.99	6.88	4.25	0.0250
NONMMUG000267.2	367	4.83	6.62	4.01	0.0310
ENSMUSG00000084925.1	745	5.45	7.08	3.62	0.0440
NONMMUG010408.2	890	3.75	5.49	3.45	0.0016
NONMMUG003195.2	996	4.52	6.15	3.37	0.0180

FC: fold change=Hypoxia group/Normoxia group

Supplementary Table 2.Top 30 down-regulated lncRNAs

LncRNA_ID	Length (bp)	Normoxia group	Hypoxia group	Log2FC	Pvalue
NONMMUG020569.2	1341	4.38	3.33	0.15	0.016
NONMMUG074505.2	10575	4.32	3.30	0.22	0.049
NONMMUG011618.2	1245	4.01	2.93	0.24	0.029
NONMMUG073129.1	508	5.52	4.45	0.24	0.020

NONMMUG039372.2	8424	3.99	2.91	0.26	0.024
NONMMUG059164	307	4.06	3.09	0.26	0.020
NONMMUG086291.1	1814	4.85	3.71	0.26	0.048
NONMMUG021861.2	2536	4.29	3.24	0.27	0.012
ENSMUSG00000099364.1	2092	4.28	3.26	0.27	0.016
NONMMUG079609.1	823	4.39	3.25	0.27	0.048
NONMMUG051365.1	1365	5.33	4.31	0.28	0.0037
NONMMUG053048.1	807	4.27	3.23	0.28	0.017
NONMMUG049312.1	1196	3.99	2.95	0.28	0.007
NONMMUG082800.1	381	4.34	3.18	0.28	0.049
NONMMUG050945.1	417	3.89	2.74	0.29	0.049
NONMMUT097816.1	1835	5.11	3.99	0.29	0.035
NONMMUG016420.2	1536	3.90	2.86	0.30	0.007
NONMMUG057217.1	695	3.82	2.76	0.30	0.012

NONMMUG053072.1	213	5.19	4.07	0.30	0.045
NONMMUG052409.1	220	3.85	2.71	0.30	0.049
NONMMUG052496.1	873	4.66	3.55	0.30	0.047
NONMMUG083353.1	3122	4.10	2.98	0.30	0.045
NONMMUG072653.1	2818	3.77	2.70	0.30	0.018
NONMMUG058127.1	288	4.59	3.44	0.30	0.045
NONMMUG005355.2	4209	4.95	3.85	0.31	0.028
NONMMUG064594.1	935	4.78	3.37	0.31	0.033
NONMMUG013304.2	2314	3.93	2.78	0.31	0.048
NONMMUG036722.2	1227	4.29	3.16	0.32	0.040
NONMMUG058787.1	1032	4.69	3.64	0.32	0.0036
NONMMUG068797.1	1440	4.30	3.18	0.32	0.030

FC: fold change=Hypoxia group/Normoxia group

Supplementary Table 3. Sequences of PCR primers used for amplification of lncRNAs

LncRNA	Forward primer	Reverse primer
NONMMUT018860	CCGACCAGCCAAATCAACA	GGTCTTTCAATCAAACCCAGTG
NONMMUT014985	TAAGAGGCAGAGGCAAGGGAT	GCACGGAGCCTAGTAAACAC
NONMMUT017709	GGGATGGCGGGATTGATG	TGGGAAGAGGCAGGTGGAG
NONMMUT100665	GGCCTGTTCTGTCTGATTCATT	TATTTGCATACACCACCACCC
NONMMUT013609	TTCAAAATCATCACAGCCAAGG	CAGCCCACAAGGGATAGGTTA
NONMMUT111660	CTGAGTGGTAGGGTCTGGTGAG	GAGTTTTGGGCTTATGGGTCTT
NONMMUT105520	CAGAGCAGGAGTTGATAGGGG	GAGCAATCATCGACTGTGCC
NONMMUT115524	GGTGGTGAATGGCTGAGGA	GGGAAAGGTGTTGTGAAAAGG
NONMMUT021455	CCCTGCTGTGCTCTACTAACCC	GAAGACTATCCCCTGGAAATGG
NONMMUT010688	AGCACCCGAAGGGAAGAAG	ATCAAACCAGCCAAGTACCAAC
NONMMUT022508	ACTCAGCCTCAGCACCCACA	GCCAACGTCAGACATCCTCTTC

NONMMUT010893	TAGTCCGGCGTTTGGGTC	TAGGTCTTTGGCAAGTGTTCG
NONMMUT092693	AGTGCTTTTGGTTCCCTTTGT	ACTTCCCACCTTCCCTCTGA
NONMMUT127165	TGTGCTTTGGGCATGGTCT	GAGGGCTTGGAACTGTCGG
NONMMUT086511	CGCTTTCCTAGTTTCCCCTTAT	AAATCCCTCACTGTGCCTTTT
NONMMUT017642	GGTATGGTTCTTGGTTTGGTCA	GATCCCTCACTTTTGGATGGTA
NONMMUT097430	AACATGAAAGTTAAGATGTGTGGC	CGGAGTGAGCAGAGGTCCTAA
NONMMUT105680	GGTTGCCAGGAAGACACGA	AGACATGATCCAGGAAGGAGATT
NONMMUT105058	ATAAAATAAGCCAACTCAAACCAAG	GCAATTTCCCTGACATCCTCC
NONMMUT121845	ACATGACTGCCCAATGTGAGTT	GGTTGAAGCATCATGGGTTTT
NONMMUT002434	GCCCTGGACCCTCTTACATT	TGAGACCTCTGAGTTCCGATTT
NONMMUT098768	TCAAGGGAGAACCACCTACAAC	GGGCTCACTTTCTCACGG

Supplementary Table 4. Information of siRNAs sequences for transfection

LncRNA-siRNA	Sense/anti-sense

LncRNA-NONMMUT021455	siRNA-1	5’ -GGAAAGTTAGACAAGAAGAA -3’/
		5’ -GGCTCATCTTATTATAGTA-3’
	siRNA-2	5’ -CAGACTTCACCTACAGTAA -3’/
		5’ -GGAGAAAGAGCGAAGTGAAG -3’
	siRNA-3	5’ -GCAGCAGAATGCAGAGGAAG -3’/
		5’ -CCTTGCAAGTCATGACAAAC -3’
Control-siRNA	siRNA-control	5’ -UUCUCCGAACGUGUCACGUTT -3’/
		5’ -ACGUGACACGUUCGGAGAATT -3’
